# Assessing the Optimal Dose of Mivacurium for Modified Rapid Sequence Intubation in Emergency Surgical Settings: A Randomized, Double-Blind Trial

**DOI:** 10.5812/aapm-153629

**Published:** 2024-11-27

**Authors:** Saad Ahmed Moharam, Ismail Mohamed Abdelgawad Ahmed, Ahmed Mahmoud Elgarhy, Sameh Hamdy Abdelhamid Seyam, Mohammed Talal Almalki, Mohammed Said ElSharkawy

**Affiliations:** 1 Anesthesiology Unit, Surgical Intensive Care and Pain Medicine Department, Faculty of Medicine, Tanta University, Tanta, Egypt; 2 Anesthesiology Unit, Intensive Care and Pain Management Department, Faculty of Medicine, Al-Azhar University, Cairo, Egypt; 3 Anesthesia Consultant, King Salman Military Hospital, Tabuk, Saudi Arabia

**Keywords:** Emergency, Mivacurium, Rapid Sequence Intubation, Dose

## Abstract

**Background:**

Modified rapid sequence intubation (RSI) is crucial in emergency surgery, particularly for patients with a full stomach, as it allows for the administration of general anesthesia (GA).

**Objectives:**

This work aimed to evaluate mivacurium effectiveness and optimal dose in modified RSI.

**Methods:**

This randomized double-blind study involved 100 patients, aged between 20 - 60 years, of both sexes, with the American Society of Anesthesiologists physical status classification of I - III, who were undergoing emergency surgery under GA. Patients were randomized into two equal groups and received mivacurium in a dose of 0.3 mg/kg in group M1 or 0.4 mg/kg in group muscarinic-2 (M2).

**Results:**

Intubating conditions were significantly better in group M2 than in group M1. The onset of adequate muscle relaxation was significantly earlier in group M2 than in group M1 (P < 0.001). At the third minute, mean arterial blood pressure recordings were significantly lower in group M2 (P = 0.04) than in group M1. The recovery time was significantly longer in group M2 than in group M1 (P < 0.001).

**Conclusions:**

Mivacurium in a 0.4 mg/kg dose resulted in more favorable intubating conditions during RSI and a more profound, earlier onset of muscle relaxation, but with a longer recovery time compared to the 0.3 mg/kg dose.

## 1. Background

Intubation of full-stomach patients undergoing emergency surgery poses a significant challenge for anesthetists. Rapid sequence intubation (RSI) is vital for alleviating the aspiration risk of gastric content (1).

Drugs such as succinylcholine and certain non-depolarizing drugs are commonly preferred due to their rapid onset of action (2).

Succinylcholine, a widely employed medication in the context of RSI, poses several risks, including hyperkalemia, cardiac arrhythmias, elevated intracranial pressure, and intraocular pressure (3). Although these medications were intended to selectively block nicotinic cholinergic receptors at the neuromuscular junction, some of the drug molecules also exhibit affinity for muscarinic cholinergic receptors located in ganglia, nerve endings, and smooth muscle. This binding can influence parasympathetic control of heart rate (HR), ultimately affecting the cardiovascular system with varying levels of potency (4).

Mivacurium Benzyl Isoquinoline, a non-depolarizing short-acting muscle relaxant, is metabolized rapidly by butyrylcholine esterases (5). Mivacurium is chosen for surgeries that require stable hemodynamics as it only causes transient tachycardia and hypotension. It has a delayed onset and does not accumulate in the body, allowing for quick recovery from muscle relaxation (6).

The typical intubation dose is 0.2 mg/kg for endotracheal intubation, after 3 minutes. A higher dose of 3 - 4 times ED95% can provide excellent rapid intubation conditions and stable hemodynamics in volume-preloaded patients (7).

The rationale for this study is to establish whether a higher dose of mivacurium (0.4 mg/kg) (8) provides better intubating conditions compared to a lower dose (0.3 mg/kg) (9), without significant side effects or hemodynamic instability.

## 2. Objectives

This work aimed to compare the 0.4 mg/kg and 0.3 mg/kg doses of mivacurium in RSI.

## 3. Methods

This randomized double-blind study involved 100 patients, aged between 20 - 60 years, of both sexes, with the American Society of Anesthesiologists physical status classification of I - III, who were undergoing emergency surgery under general anesthesia (GA). The research was conducted between July and November 2023, after obtaining the necessary approval from the Ethical Committee of Al-Azhar University Hospitals (approval code: 0354/2023) and registration on clinicaltrials.gov (ID: NCT06072534). The patients provided informed written consent. The study design followed the guidelines of the Consolidated Standards of Reporting Trials (CONSORT).

Exclusion criteria included morbid obesity, succinylcholine apnea, pregnancy, decompensated heart disease, anticipated difficult airway, severe pulmonary disease, allergy to any of the induction agents, and known neuromuscular disease.

### 3.1. Randomization and Blinding

A computer-generated randomization process was employed to allocate patients to either of the two groups. Patients' codes were placed in an opaque, sealed envelope to maintain the concealment of the allocation. Patients were randomized to one of the two groups in a 1: 1 ratio, with equal numbers of patients in each group. Following randomization, patients received mivacurium in a dose of 0.3 mg/kg in group M1 and 0.4 mg/kg in group muscarinic-2 (M2). The patient and the anesthesiologist performing the intubation were blinded to the specific dose of medication that was injected.

All patients underwent history taking, clinical examination, and laboratory investigations. All patients were monitored using pulse oximetry, noninvasive blood pressure, temperature probe, electrocardiogram, and end-tidal CO_2_ and received Ringer’s solution preload. Proper preoxygenation was performed, followed by the induction of GA with propofol 2 mg/kg. The assistant anesthetist administered lidocaine at 1.5 mg/kg, fentanyl at 1 µg/kg, and mivacurium at a dose of either 0.3 mg/kg or 0.4 mg/kg of the ideal body weight according to grouping. Tracheal intubation was performed by the same anesthetist using a Macintosh laryngoscopy blade.

Intubation conditions were assessed using the Cooper score of 10 (10), 90 seconds after administering the muscle relaxant. The total score was calculated and categorized as follows: Zero - 2 (poor), 3 - 5 (fair), 6 - 7 (good), and 8 - 9 (excellent) (Table 1). 

**Table 1.** Criteria and Score of Intubation Conditions (Cooper Score)

Score	Jaw Relaxation	Vocal Cords	Response to Intubation
**0**	Poor (impossible)	Closed	Severe coughing/bucking
**1**	Minimal (difficulty)	Closing	Mild cough
**2**	Moderate (fair)	Moving	Slight diaphragmatic movement
**3**	Good (easy)	Open	None

An additional propofol 0.5 mg/kg was administered if poor or fair score conditions were observed. Isoflurane maintained anesthesia at 1.2%.

Heart rate and noninvasive mean arterial pressure (MAP) were recorded before and after intubation for 5 minutes at 1-minute intervals.

Train of four (TOF) was monitored using a TOF scan (Drager technologies, Canada acceleromyography monitors) every five minutes until recovery from relaxation effect and appearance of T1 first twitch. No reversal drugs were required.

Side effects such as breathing problems, eye irritation, and itching were documented.

The primary outcome was to assess the intubation condition. The secondary outcomes were hemodynamic changes during and after intubation, time of recovery, and side effects.

### 3.2. Sample Size Calculation

The sample size calculation was performed using G*Power 3.1.9.2 (Universität Kiel, Germany). Before the main study, a pilot study was conducted with 10 cases in each group, which revealed that the incidence of excellent intubating conditions was 30% in group M1 and 60% in group M2. Based on these findings, we employed a sample size calculation with the following parameters: 95% confidence limit, 80% power of the study, a group ratio of 1: 1, and an additional eight cases in each group to account for potential dropouts. This calculation resulted in a required sample size of 50 patients per group.

### 3.3. Statistical Analysis

Data collection and analysis were performed using SPSS version 21 for Windows (SPSS Inc., Chicago, Illinois, USA). Normally distributed quantitative variables were presented using mean ± standard deviation, while frequencies and percentages were used to display qualitative data. To compare normally distributed quantitative variables, an independent *t*-test was employed. For qualitative data, a chi-square (χ²) test was used, unless the cell count was < 5, in which case the Fisher exact test was applied. The chosen level of statistical significance was set at P < 0.05.

## 4. Results

**Figure 1.**
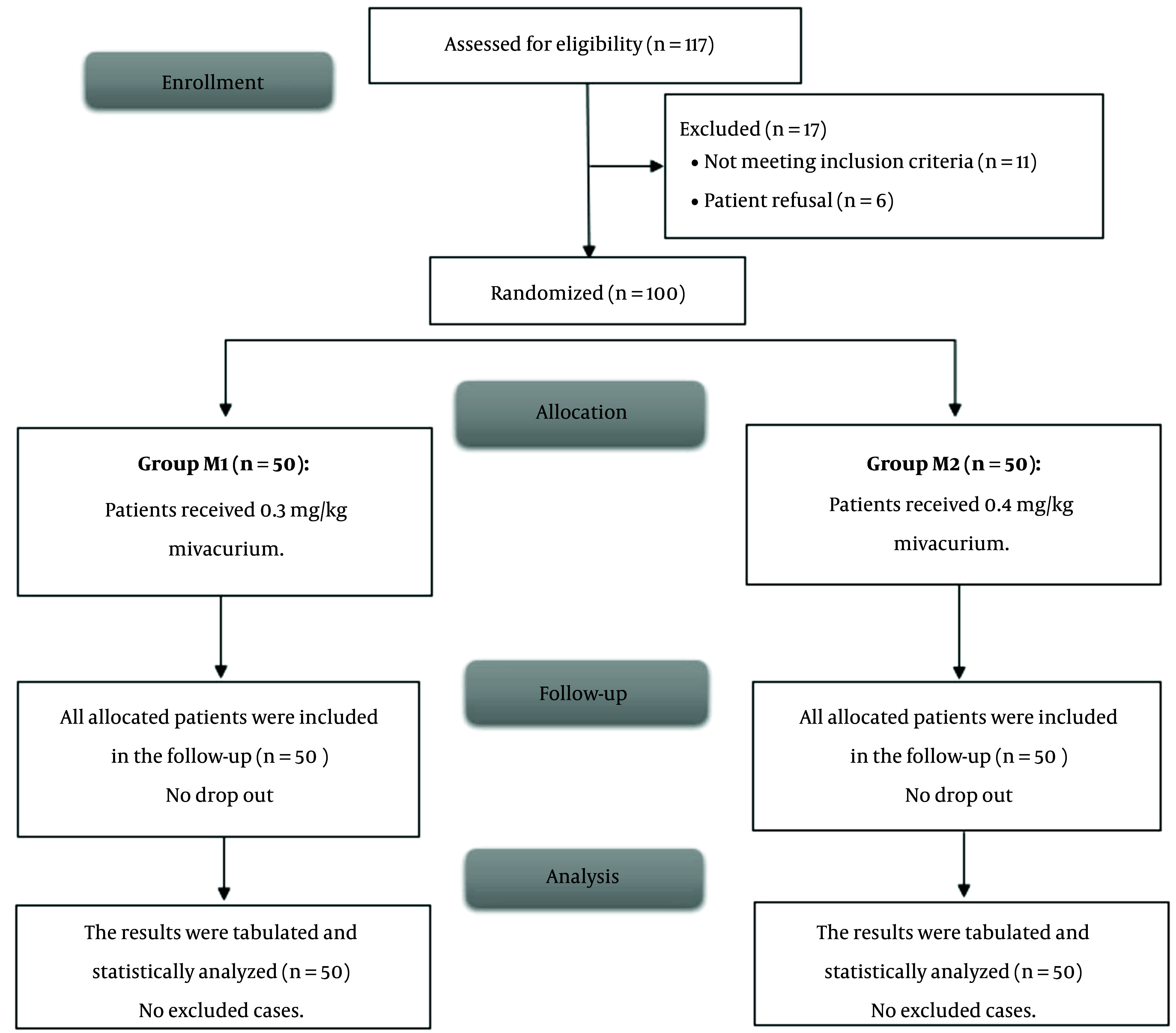
CONSORT flow chart of the studied cases

In this trial, 117 patients were initially screened for eligibility to participate in this study, from whom 11 did not meet the inclusion criteria and 6 declined to participate. The remaining 100 patients were then randomly assigned to two groups of equal size. All patients who were allocated to the study were subsequently followed up and included in the statistical analysis (Figure 1). 

Demographic data were comparable between both groups, M1 and M2 (P > 0.05) (Table 2). 

**Table 2.** Demographic Data of Studied Patients ^a^

Variables	Group M1 (N = 50)	Group M2 (N = 50)	P	CI 95%
**Age, (y)**	36.98 ± 9.16	36.52 ± 7.79	0.787	-2.92: 3.84
**Sex**			0.230	0.85: 1.95
Male	27 (54)	21 (42)		
Female	23 (46)	29 (58)		
**Weight, (kg)**	71.92 ± 7.82	75.12 ± 8.73	0.056	-6.49: 0.09
**ASA**			0.671	0.74: 1.42
I	28 (56)	31 (62)		
II	20 (40)	16 (32)		
III	2 (4.0)	3 (6.0)		

Abbreviation: ASA, American Society of Anesthesiologists.

^a^ Data is presented as mean ± SD or frequency (%).

Intubating conditions were significantly superior in group M2 compared with group M1. In group M2, 64% of the patients had excellent intubation conditions, while in group M1, only 12% had similar conditions. Fair conditions were only recorded in group M1, with 32% of these patients, and no patients in group M2 experienced fair intubating conditions. No poor intubating conditions were reported in either of the groups (Table 3). 

**Table 3.** Intubating Conditions of the Studied Patients Undergoing Modified Rapid Sequence Intubation ^a^

Variables	Group M1 (N = 50)	Group M2 (N = 50)	P	CI 95%
**Excellent**	6 (12)	32 (64)	< 0.001 ^b^	0.45: 2.17
**Good**	28 (56)	18 (36)		
**Fair**	16 (32)	0 (0)		
**Poor**	0 (0)	0 (0)		

^a^ Data is presented as frequency (%).

^b^ Significant as P < 0.05.

**Figure 2.**
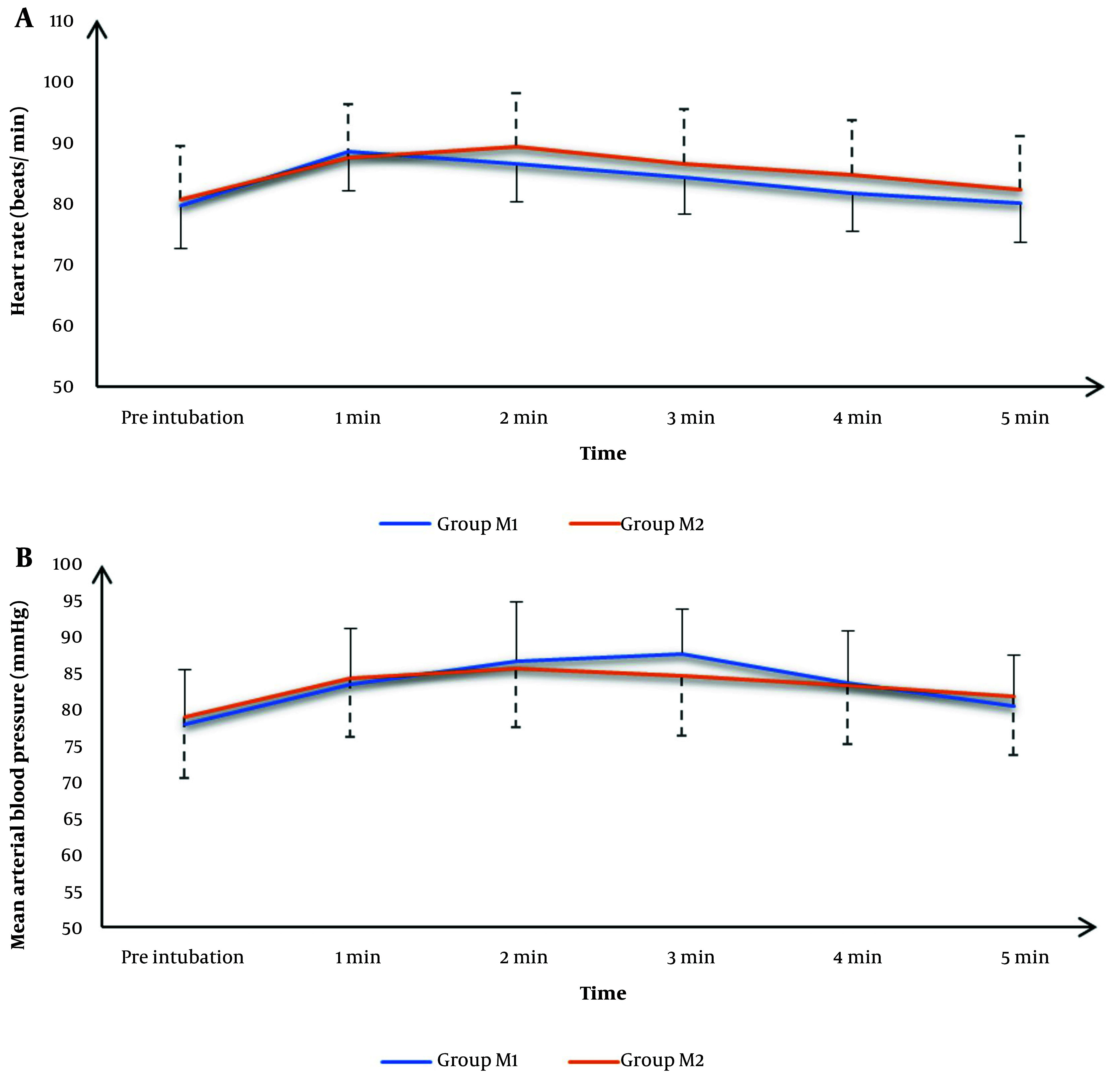
A, heart rate (HR); and B, mean arterial pressure (MAP) changes of the studied groups

Recorded HR readings preoperatively, after 1 minute, 2 minutes, 3 minutes, 4 minutes, and 5 minutes were statistically comparable between both groups (P > 0.05) (Figure 2A ).

The mean MAP values were significantly lower in group M2 at 3 minutes than in group M1 (P = 0.04); otherwise, mean MAP values were comparable between both groups (P > 0.05). (Figure 2B )

Patients who required propofol were comparable between both groups. The onset of adequate muscle relaxation and time to recovery were significantly earlier in group M2 than in group M1 (P < 0.001) (Table 4). 

**Table 4.** Patients Required Additional Propofol, Onset of Adequate Muscle Relaxation, Needed Reversal of the Neuromuscular Blocking Effect and Time to Recovery of the Studied Groups ^a^

Variables	Group M1 (N = 50)	Group M2 (N = 50)	P	CI 95%
**Patients required additional propofol**	6 (12)	1 (2)	0.111	0.74: 48.04
**The onset of adequate muscle relaxation (sec)**	141.52 ± 18.01	120.62 ± 20.1	< 0.001 ^b^	-28.47: -13.32
**Patients needed reversal of the neuromuscular blocking effect**	0 (0)	0 (0)	-	-
**Time to recovery (min)**	32.5 ± 2.5	42.6 ± 2.5	< 0.001 ^b^	9.108: 11.092

^a^ Data is presented as frequency (%) or mean ± SD.

^b^ Significant as P < 0.05.

No side effects were recorded, including breathing problems, eye irritation, or itching.

## 5. Discussion

Frequent adverse effects of depolarizing neuromuscular blockers and the need for neuromuscular reversal drugs have been significant factors that have led to the requirement for non-depolarizing neuromuscular blocking agents (11). Mivacurium has been preferred in certain situations to facilitate spontaneous neuromuscular function recovery. However, the delayed onset of action posed a significant challenge to its use (12).

In this study, patients who were administered mivacurium at a dosage of four times the effective dose 95 (ED95) in group M2 had a significantly higher occurrence of acceptable intubation conditions (100%) compared to group M1, where patients received the drug at a dosage of three times the ED95 (68%). Holkunde et al. (13) found that optimal conditions for intubation were achieved in 56.7% of cases when administering atracurium at a dosage of 4 times the ED95 of 1 mg/kg. In contrast, only 13.3% of cases achieved excellent intubating conditions when receiving atracurium at a dosage of more than three times the ED95 (0.75 mg/kg). According to Shanks et al. (14), 75% of the participants achieved excellent intubation conditions when using 2.5 mg/kg of mivacurium, compared to only 47.2% who used a lower dose of 1.5 mg/kg. These results were observed along with the administration of thiopental and fentanyl.

Similarly, Tang et al. (15) found that 72% of patients achieved satisfactory intubation when administered 0.2 mg/kg of mivacurium. Additionally, the use of reversal drugs was significantly eliminated. They suggested that administering higher doses of mivacurium (0.25 mg/kg) could enhance intubating conditions even more. Vanacker et al. (16) found that 25% of the patients studied had excellent intubation conditions when using 0.2 mg/kg of mivacurium. In contrast, Turkmen et al. (17) reported that 55% of the patients had excellent conditions when using 0.25 mg mivacurium. The two studies mentioned above utilized propofol, along with fentanyl and alfentanil, respectively. Vanlinthout et al. (18) conducted a meta-analysis on 1050 healthy subjects from 29 studies. They found that injecting a higher dose of mivacurium, up to 0.27 mg/kg, was one of the key factors contributing to achieving excellent intubating conditions.

In contrast, Dieck et al. (19) terminated the study early due to poor intubating conditions on induction 3 minutes after 0.2 mg/kg of mivacurium, 2.5 mg/kg of propofol, and 1 µg/kg of remifentanil.

In the current study, the HR showed comparable recordings in both groups, M1 and M2. A significant decrease in MAP after the 3rd minute in group M2 was detected in this study. This is consistent with Okanlami et al. (20), who found that mivacurium in a dose of (10^-9^ - 10^-5^ mmol/kg) inhibited bradycardia. This finding is consistent with Savarese et al. (21) and Savarese (22), who noted that mivacurium administration was associated with a temporary reduction in blood pressure. They attributed this to mivacurium's ability to block both M2 and M3 receptors, with equal potencies for both receptors.

The study also found a significant increase in the recorded TOF in group M2 compared to group M1 (P < 0.001). Additionally, no reversal drugs were required, which aligns with the findings of Tang et al. (15).

Vested et al. (23) reported that there was no significant disparity in the timing of onset between mivacurium dosages of 0.2 mg/kg administered to elderly and younger patient populations. However, the study revealed that the duration of mivacurium's effects was significantly prolonged in the elderly patient group. Furthermore, both groups did not exhibit any statistically significant differences in terms of intubating conditions.

The limited dose range was also considered a limitation in the study, as we only evaluated two doses of mivacurium (0.3 mg/kg and 0.4 mg/kg), which may not represent other doses or dosing regimens. The study was conducted over a short period, which may not provide a comprehensive understanding of the long-term effects or optimal dosing of mivacurium in RSI. The study only evaluated the use of mivacurium and did not compare its effectiveness to other commonly used muscle relaxants in RSI. Mivacurium has experienced intermittent unavailability in recent times. The study involved a specific age group (20 - 60 years) and physical status classification (ASA I-III), which may limit applicability to older patients or those with more severe comorbidities. Patients with conditions such as morbid obesity, severe pulmonary disease, and known neuromuscular diseases were excluded, which may limit generalizability. The single-center nature of the study may limit external validity. To enhance generalizability, future studies should consider larger and more diverse patient populations, compare mivacurium with other agents, and explore the impact of various comorbidities on intubation conditions.

### 5.1. Conclusions

Mivacurium in a 0.4 mg/kg dose resulted in more favorable intubating conditions during RSI but with a delayed spontaneous recovery time. Mivacurium in 0.4 mg/kg resulted in a more profound and earlier onset of muscle relaxation.

## Data Availability

Data is available upon resonable request from corresponding author.

## Clinical Trial Registration Code:

NCT06072534.
